# Occupational Health Hazards Among Veterinarians in Saudi Arabia

**DOI:** 10.7759/cureus.47822

**Published:** 2023-10-27

**Authors:** Sanad Al-Harbi, Ali Al-Doweriej, Mohamed Aljaser, Sara Abdulrahman, Omar S Alnuwais, Sara M Nader, Hussein Lulu, Ahmed S Abdel-Moneim, Manal S Hussein, Azza H Abd-El-Rahman, Samy Kasem

**Affiliations:** 1 Animal Health Sector, National Center Prevention & Control of Plants Pests & Animal Diseases, Riyadh, SAU; 2 Department of Zoonoses, Faculty of Veterinary Medicine, Cairo University, Giza, EGY; 3 Animal Health Sector, Ministry of Environment, Water and Agriculture, Riyadh, SAU; 4 Department of Microbiology, College of Medicine, Taif University, Taif, SAU; 5 Department of Pharmacology, College of Medicine, Taif University, Taif, SAU; 6 Department of Pathology, College of Medicine, Taif University, Taif, SAU; 7 Department of Virology, Faculty of Veterinary Medicine, Kafrelsheikh University, Kafr El Sheikh, EGY

**Keywords:** middle east respiratory syndrome coronavirus (mers-cov), viruses, q-fever, leishmaniosis, toxoplasma gondii infection, mycobacterium tuberculous, brucella species, fmdv, avian influenza, occupational diseases

## Abstract

Introduction

Veterinarians and other professionals who interact with animals on a daily basis encounter an elevated risk of exposure to both known and as-yet-undiscovered microbial agents. Additionally, they are also exposed to physical, chemical, and environmental hazards. Enhancing occupational health and safety in this context carries significant global significance.

Methods

This study aimed to comprehensively identify and outline the various biological, physical, chemical, and environmental health threats that were encountered by veterinarians in Saudi Arabia. To achieve this, we designed a self-completed questionnaire for 529 participants. The survey encompassed potential occupational hazards such as microbial diseases, injuries resulting from animal bites and scratches, allergies, and environmental risks like sunstroke and dust storms.

Results

Among the 529 participating veterinarians, 45.9% (243 individuals) reported instances of zoonotic diseases within the past five years. Notably, potential viral agents included Middle East respiratory syndrome coronavirus, avian influenza, and foot-and-mouth disease virus. Bacterial diseases were also frequently documented, with brucellosis (18.7%) and salmonellosis (7.9%) being notable pathogens. Protozoal infections were led by Leishmaniosis, constituting the most commonly detected protozoa (29 /529, 5.5%). Interestingly, 345 (65.2%) of the individuals reported that they have experienced animal bites and scratches. Needle stick injuries were also a common occupational hazard, with an incidence rate of 19.1%. Additionally, chemical exposure was prevalent, particularly to disinfectants (57.5%) and veterinary drugs (23.4%). The study participants also reported their exposure to various environmental hazards, including sunstroke, dust, sandstorms, and heavy rains.

Conclusion

The findings of this study draw attention to a concerning trend among veterinarians in Saudi Arabia. Their personal health and safety appear to receive inadequate attention, potentially heightening the risk of occupationally related health hazards. These outcomes highlight the need for a reevaluation of safety protocols and infection control practices within the veterinary profession. The implications of this study can potentially inform the development of policies and initiatives aimed at mitigating occupationally related health hazards among veterinarians in Saudi Arabia.

## Introduction

The influence of veterinarians extends beyond animal health alone, as their work is instrumental in safeguarding human health and the environment. This comprehensive approach involves not only the prevention and management of animal diseases but also the mitigation of zoonotic diseases, which can be transmitted from animals to humans. Additionally, veterinarians play a crucial role in ensuring the safety and quality of food products originating from animals [1]. Veterinarians hold diverse roles that encompass a wide range of domains, from the care and well-being of animals to ensuring food safety and safeguarding public health. Those specializing in the care of food animals are responsible for diagnosing and treating illnesses and injuries among these animals, conducting disease testing, and administering vaccinations. Veterinarians are exposed to occupational hazards characteristic of healthcare professions [1,2]. In food inspection and safety, along with those involved in veterinary public health, they are deeply concerned with every aspect of the food production chain. Their responsibilities span disease prevention measures, carcass inspection, and the scrutiny of food products before they reach store shelves. In this capacity, they are tasked with conducting rigorous checks on animal products imported from abroad, thereby ensuring the integrity of our food supply. Furthermore, veterinarians working in laboratory settings perform diagnostic procedures across various specialized fields, contributing to the advancement of veterinary science and healthcare [2].

Veterinarians interact with a variety of animals and birds, some of which may carry infectious agents that pose a risk of zoonotic disease transmission to veterinarians [2]. Occupational zoonotic diseases are among the most common occupational health hazards. Zoonotic diseases place a significant burden on the economy of any country, with a more pronounced impact on developing and poorly developed nations. Globally, according to the World Health Organization (WHO), zoonotic diseases result in one billion cases of illnesses and countless deaths annually [3].

In the past, the primary risk of occupational diseases was associated with zoonotic infections such as viral, bacterial, mycotic infections, protozoal infections, and parasitic infections. Additionally, injuries, radiation exposure, chemical exposure, and allergies contribute significantly to a broad spectrum of professional risks. Present-day risks encompass new factors, including mental and physical stress [4,5]. Veterinarians are exposed to various occupational health risks, with over 40% of participants reporting a zoonotic disease at some point in their careers [6]. The reported zoonotic diseases in Western Canada included brucellosis, rabies exposure, animal bites, psittacosis, erysipelothrix infection, and leptospirosis [6]. In another study, a serological survey unveiled that the most common zoonotic infections in Australia were brucellosis, toxoplasmosis, and Q-fever [6,7]. While veterinarians are at an elevated risk of contracting zoonotic infections, the occurrence of specific zoonoses in humans depends on factors such as the frequency of infection in local animal populations, the likelihood of disease transmission, the availability and use of personal protective equipment, and even the quality of veterinary education [8].

On the other hand, veterinarians working in large animal and mixed practices are more likely to encounter occupational risks related to musculoskeletal injuries, needle sticks, cuts, and animal bites, given their regular treatment of a significant number of companion animals such as dogs and cats [9]. Australian researchers have identified animal-related injuries, particularly dog and cat bites, as important hazards in the veterinary profession [10]. Due to the fact that many species carry pathogens in their mouths, animal bites can result in conditions such as cellulitis, abscesses, and more severe complications, including sepsis, arthritis, endocarditis, and central nervous system (CNS) infections, including rabies [6].

Occupational allergies may also develop among veterinarians, often triggered by contact with vaginal secretions, amniotic fluids, or latex gloves and exposure to blood proteins or parasites [10]. Reports of occupational asthma have also emerged; a survey of veterinarians in the Netherlands found that, after accounting for smoking status, the odds of chronic cough and phlegm production were higher in veterinarians working more than 20 hours per week in a swine facility [11].

Previous research has explored the occupational health risks faced by veterinarians in Saudi Arabia. The findings underscored the need for improved safety measures in veterinary practice. These earlier research efforts have contributed to our understanding of the unique health hazards veterinarians in this region face, and they have laid the groundwork for further investigations into occupational health and safety for veterinarians in Saudi Arabia and the Arabian Peninsula [12,13].

The objective of this study was to determine the prevalence of occupational hazards, including zoonotic, physical, chemical, and environmental hazards, among veterinarians in Saudi Arabia.

## Materials and methods

Study design

A cross-sectional cohort was designed to assess the occupational health hazards that have been encountered by veterinarians and veterinary technicians in Saudi Arabia. Data was collected using an online-based questionnaire that has been approved by the Ministry of Environment, Water, and Agriculture (MEWA) ethical committee (MEWA1443/07).

Subjects

The sample size for this study was determined using the RaoSoft tool (Raosoft Inc., Seattle, WA), a free online tool for sample size calculation. A confidence level of 99% and a margin of error set at 5%, were used. The inclusion criteria for this study were intentionally defined to encompass individuals classified as veterinarians or veterinary technicians who had engaged in professional activities involving animals over the preceding five years. This specific criterion ensured that the respondents possessed relevant professional experience and insights into the occupational risks and health hazards faced by those within the veterinary field. Throughout the study, clear instruction was demonstrated to the potential participants that their involvement was entirely voluntary. Completing the questionnaire was explicitly regarded as informed consent, and there was no obligation for any individual to participate.

Study duration and communication

This research study spanned a duration of one month, commencing on March 1st, 2022. An online questionnaire was distributed by WhatsApp groups and email communication as the primary modes of engagement due to its inherent efficiency and wide-reaching accessibility. It included a comprehensive introduction to the study's objectives. In the online questionnaire, a concise yet informative overview was provided about the prevalent zoonotic diseases in the Kingdom of Saudi Arabia (KSA) and highlighted specific chemical, physical, and environmental hazards of particular concern to veterinarians within the KSA context.

Questionnaire content

The questionnaire was initiated by soliciting basic demographic information, encompassing details such as participants' nationality, age, gender, place of employment, and the primary animal species they typically attended to. Following there were four open-ended questions, each designed to thoroughly assess confirmed exposures to microbial pathogens, chemical agents, physical factors, and environmental health hazards. 

Statistical analysis

The initial phase of data processing involved the meticulous coding and categorization of the quantitative data using Microsoft Excel 365. Each data point was systematically assigned to its respective category, ensuring precision and consistency in subsequent analyses. Following this, the dataset underwent thorough analysis using IBM SPSS Statistics for Windows, Version 16 (Released 2007; IBM Corp., Armonk, New York, United States). Both the Chi-square test and Fisher's exact test were employed in Crosstabs to assess the significance of differences among groups. Fisher's exact test was applied when the expected cell count was less than 5. A significance level (p< 0.05) would typically be determined to evaluate whether the observed associations are statistically significant. The categorical variables were expressed as numbers and percentages.

## Results

Demographic data

Five hundred twenty-nine participants, comprising five females (0.9%) and 524 (99.1%) males, submitted their responses to the designed questionnaire. This group included 457 (86.4%) Saudi residents and 72 (13.6%) non-Saudi residents. The participants consisted of 91 (17.2%) veterinary assistants, 370 (69.9%) veterinarians, 55 (10.4%) veterinarians holding master's degrees, and 13 (2.5%) holding PhDs. The majority of participants (503, 95.1%) primarily had contact with food animals such as sheep, goats, camels, cattle, and poultry. Meanwhile, participants in contact with equine or companion animals constituted 2.3% and 2.6% of the total, respectively (Table 1).

**Table 1:** Demographic data of the participants. *The first bachelor’s degree is Veterinary Medicine.

Variable	Item	Number	Percentage
Age	20-30	131	24.8
	31-40	238	45
	41-50	119	22.5
	51-60	41	7.8
Sex	Female	5	0.9
	Male	524	99.1
Education	Diploma (Veterinary assistant)	91	17.2
	Bachelor of Veterinary Medicine	370	69.9
	Master *	55	10.4
	PhD *	13	2.5
Nationality	Saudi	457	86.4
	Non-Saudi	72	13.6
Employer	Governmental sector:	467	88.3
	Private sector	62	11.7
Primary Species	Food animals (sheep, goat, camels, cattle, and poultry)	503	95.1
	Equine	12	2.3
	Companion Animals	14	2.6

Zoonotic hazards

A total of 243 out of 529 participants (45.9%) reported individual exposure to different pathogens. In the current study, three viral diseases were reported among the participants, including 10 cases of MERS-CoV, six cases of avian influenza, and three cases of FMDV (Table 2). Bacterial infections were reported more frequently than viral infections among the total participants, with instances of brucellosis (99, 18.7%), salmonellosis (42, 7.9%), tuberculosis (3, 0.6%), Q fever (4, 0.8%), and chlamydiosis (3, 0.6%). Fungal infection was reported in 21 cases (4.0%) among the total participants. Leishmaniosis was the most frequently reported protozoal infection (29, 5.5%), whereas toxoplasmosis and giardiasis were rarely reported, with only two cases (0.4%) and one (0.2%) case, respectively (Table 2). Significant variation among different age groups was reported for both brucellosis and leishmaniosis (Table 2). Furthermore, exposure to animal bites implies a possible risk of rabies virus (Table 3).

**Table 2:** Prevalence of biological hazards among Saudi veterinarians and veterinary technicians. *Significant variation among different age groups was reported for both brucellosis (P<0.002) and leishmaniosis (P<0.043). Both Chi-square and Fisher's exact test were used to determine the significant difference among groups. Fisher's exact was used to determine the significance when the expected count was less than 5. MERS-CoV: Middle East respiratory syndrome coronavirus

	Infection	Sex		P value	Age				P value	Cumulative	
		Male	Female		20-30	31-40	41-50	51-60			
										Number	%
MERS-CoV	Yes	9	1	0.142	1	0	1	2	0.97	10	1.9
	No	512	4		130	238	116	39		519	98.1
Avian influenza	Yes	5	1	0.912	3	3	0	0	0.326	6	1.1
	No	519	4		128	235	117	41		523	99.9
Foot and mouth disease	Yes	3	0	0.955	1	1	1	0	0.724	3	0.6
	No	521	5		130	237	116	41		526	99.4
Brucellosis	Yes	98	1	0.529	10	53	27	11	0.002*	99	18.7
	No	426	4		121	185	91	31		430	81.3
Salmonellosis	Yes	40	2	0.157	5	10	3	3	0.214	42	7.9
	No	484	3		126	228	114	38		487	92.1
Tuberculosis	Yes	3	0	0.955	0	3	0	0	0.902	3	0.6
	No	521	5		131	235	117	41		526	99.4
Q-fever	Yes	4	0	0.941	0	2	0	0	0.951	4	0.8
	No	520	5		131	236	117	41		525	99.2
Chlamydiosis	Yes	3	0	0.725	1	2	0	0	0.955	3	0.6
	No	521	5		130	236	117	41		526	99.4
Ringworm	Yes	21	0	0.845	2	4	2	0	0.087	21	4
	No	503	5		129	234	115	41		508	96
Leishmaniosis	Yes	28	1	0.85	1	12	6	2	0.043*	29	5.5
	No	496	4		130	226	111	39		500	94.5
Toxoplasmosis	Yes	2	0	0.97	1	1	0	0	0.766	2	0.4
	No	522	5		130	237	117	41		527	99.6
Giardia	Yes	1	0	0.985	0	1	0	0	0.381	1	0.2
	No	523	5		130	238	117	41		528	99.8

**Table 3:** Prevalence of physical, chemical, and environmental hazards among Saudi veterinarians and veterinary technicians. Both Chi square and Fisher's exact test were used to determine the significant difference among groups. Fishers’ exact test was used to determine the significance when the expected count was less than 5.

Hazard	Variable	Response	Sex		P value	Age				P value	Cumulative	
			Male	Female		20-30	31-40	41-50	51-60		Number	Percentage
Physical	Bite and scratching	Yes	341	5	0.565	85	149	82	29	0.576	345	65.2
		No	183	0		46	89	37	12		184	34.8
	Butting and kicking	Yes	347	5	0.533	89	151	83	30	0.549	353	66.7
		No	176	0		42	87	36	11		176	33.3
	Accidental cuts or punctures with needles	Yes	101	0	0.181	29	38	27	7	0.324	101	19.1
		No	423	5		102	200	92	34		428	80.9
Chemical	Allergy from pesticides	Yes	153	0	0.063	43	63	36	11	0.578	153	28.9
		No	371	5		88	175	83	30		376	71.1
	Allergy from veterinary drugs	Yes	124	0	0.116	37	47	33	7	0.123	124	23.4
		No	400	5		94	191	86	34		405	76.6
	Chemical burns	Yes	88	0	0.230	27	31	24	6	0.170	88	16.6
		No	436	5		104	207	95	35		441	83.4
	Skin allergy	Yes	300	4	0.295	77	132	70	25	0.864	304	57.5
		No	224	1		54	106	49	16		225	42.5
	Allergy from using disinfectants	Yes	133	0	0.096	38	52	34	9	0.322	133	25.1
		No	391	5		93	186	85	32		396	74.9
	Eye allergy from chemicals	Yes	200	2	0.716	57	80	49	16	0.274	202	38.2
		No	324	3		74	158	70	25		327	61.8
Environmental	Sunstroke	Yes	367	5	0.98	94	160	85	33	0.350	372	70.3
		No	157	0		37	78	34	8		157	29.7
	Sheds dust	Yes	436	5	0.363	113	191	101	36		441	83.4
		No	88	0		18	47	18	5		88	16.6
	Sandstorms	Yes	326	5	0.96	83	141	78	29	0.432	331	62.6
		No	198	0		48	97	41	12		198	37.4
	Heavy rain	Yes	244	4	0.729	66	104	58	20	0.637	248	46.9
		No	280	1		65	134	61	21		281	53.1

Physical hazards

Needle-stick injuries were the most frequently reported physical health issues among veterinarians. Needle stick injuries accounted for an average of 19.1% (101/529) of practicing veterinarians. The prevalence of occupational animal bites and scratches among veterinarians in Saudi Arabia was 65.2% (345/529). Notably, pet animal bites constituted the majority of animal bite cases (data not shown). Furthermore, animal butting and kicking were also commonly reported in large animal practices, with 66.7% (353/529) of all responding veterinarians in Saudi Arabia having experienced such injuries (Table 3).

Chemical hazards

Veterinarians were frequently exposed to various chemicals, including drugs, disinfectants, antiseptics, acaricides, liquid nitrogen, waste anesthetic gases, corrosive agents, pesticides, and more. Among these, disinfectants like sodium hypochlorite, hydrogen peroxide, and phenol posed chemical health hazards to approximately 57.5% of veterinarians. Furthermore, 23.4% of veterinarians reported experiencing the toxic effects of veterinary drugs. Another significant group of chemical health hazards included pesticide compounds, which were prevalent in an era of vector-rich populations, with around 29% of responding veterinarians being exposed (Table 3).

Environmental hazards

Among the 529 veterinarians who responded to the survey question about environmental hazards, 70% (372) reported personal experiences with sunstroke. Additionally, approximately 83.4% (441) had encountered issues related to shed dust, and 62.6% (331) had been affected by sandstorms. Environmental hazards also included heavy rains, with 56% and 46.9% of participants reporting exposure to these conditions, respectively (Table 3).

## Discussion

In this study, we focused on the occupational health hazards among veterinary medicine professionals in Saudi Arabia to explore the zoonotic, physical, chemical, and environmental hazards related to this occupation. Biological risks are considered the main occupational risk for vets. They are present in all work activities that involve exposure to biological agents. Zoonoses proved to be the most frequent cause of occupational diseases in veterinary practice [14]. The present study has documented several zoonotic diseases among the participating individuals. The outcomes resulting from exposure to the most prevalent zoonotic agents can encompass a broad spectrum, ranging from mere seroconversion to diseases exhibiting highly variable symptomatic manifestations. Furthermore, such exposure may lead to the development of irreversible sequelae or even result in fatality [2].

A total of 243 out of 529 participants (45.9%) reported individual exposure to different pathogens including MERS-CoV, cases of avian influenza (AI), FMDV, brucellosis, salmonellosis, tuberculosis, Q fever, chlamydiosis, ringworm, leishmaniosis, toxoplasmosis, and giardiasis. The results in our study are similar to a survey conducted by Dowd et al. in 2013 [15] among Australian veterinarians, wherein 44.9% reported acquiring a zoonotic disease throughout their career while differing from a survey conducted in India, where only 27% of the veterinarians were subjected to zoonotic diseases. This low percentage is likely attributed to the fact that most Indian veterinarians did not undergo zoonotic disease screening [16]. Compared to individuals practicing in other veterinary practices, farm animal veterinarians (large animal veterinarians) have a threefold higher risk of contracting zoonotic infections [16].

Human infections with FMDV are exceptionally rare and reported only in three cases in the current study. Human FMDV infection is usually associated with direct exposure to infected animals or contaminated materials in regions where FMD is endemic. Veterinarians, abattoir workers, and farmers who work closely with livestock are at a higher risk of FMDV exposure due to their occupational activities. Human FMDV infections typically manifest as a mild and self-limiting illness. Common symptoms include fever, sore throat, and the development of vesicular lesions on the hands, feet, and oral mucosa [17,18].

Both H5N1 and H9N2 were reported in Saudi Arabia [19,20] and raised significant concerns due to their zoonotic potential and potential for human-to-human transmission. Veterinarians working with poultry and in high-risk areas, where AI is prevalent in birds, face an elevated risk of exposure to AIV viruses due to their close contact with infected birds. The current study reported six cases of AI virus infection among Saudi veterinarians; however, the particular subtype was not reported.

MERS-CoV is a zoonotic virus that primarily infects dromedary camels and can be transmitted to humans. MERS-CoV infections in humans may range from asymptomatic or mild respiratory symptoms to severe pneumonia with acute respiratory distress syndrome with fatal consequences [21-23]. Veterinarians working with camels or in regions where MERS-CoV is endemic may be at risk of exposure. While human-to-human transmission of MERS-CoV has occurred, it is typically limited [24]. The risk of veterinarians transmitting the virus to others is low; however, a considerable number of MERS-CoV cases were reported in 10 cases among veterinarians and veterinary technicians in the current study.

In the current study, it was evident that veterinarians frequently encounter animals in their daily work, increasing their susceptibility to animal bites. Notably, pet animal bites constituted the majority of animal bite cases, accounting for 76-94% of total animal bites worldwide. This occupational risk is particularly relevant when dealing with aggressive or frightened animals [25]. Although rabies was not reported in the current study, rabies is a zoonotic disease transmitted through the saliva of infected animals via bites or scratches. Veterinarians are at risk of rabies exposure when handling or examining animals with potential rabies exposure history [26].

Brucellosis is a major zoonosis that continues to be of public health and economic concern in many parts of the world. In our study, the most common occupational zoonotic disease that occurs among those dealing with animals is brucellosis, with 46%, and this finding is explained by Meky et al. [27], who reported that workers in occupations dealing with animals had a 2.4-fold higher risk of brucellosis than those in occupations not dealing with animals in Iran [28]. Similarly, brucellosis was reported among 99 (18.7%) veterinarians and veterinary technicians in the current study. The most affected occupations with brucellosis are butchers and abattoir workers, most likely because of regular handling of sharp objects and intimate contact with potentially contaminated animals and their organs. Airborne and conjunctival routes of infections were thought to be essential in brucellosis transmission among this group [29], especially in closed areas such as slaughterhouses where direct contact with contaminated viscera and secretions occurs. The risk was increased when preventive measures were not properly implemented, as seen by the poor use of personal protective equipment (PPE) such as gloves, masks, goggles, boots, and aprons. Potential shortcomings in PPE usage underscore the importance of ongoing education, improving PPE comfort and accessibility, and fostering a safety-focused culture within the veterinary community. Veterinarians should understand that the proper use of PPE lets them be able to protect themselves, thus reducing the risk of zoonotic disease transmission. In Egypt, veterinary personnel had the highest percentage of seropositivity (46.2%), followed by butchers (23.1%) and employers (7.7%) [30].

Several studies have investigated the prevalence of zoonotic Salmonella infections in both animals and humans in Saudi Arabia, indicating the potential risks faced by animal workers, including veterinarians. Al-Mazrou in 2004 reviewed food poisoning in Saudi Arabia [31]. Salmonella was found to be a potential cause of food poisoning [31]. Additionally, zoonotic Salmonella serovars in poultry farms are the most common source of infection as a foodborne pathogen [32]. These studies collectively underscore the significance of zoonotic Salmonella infections in animal-related occupations in the region. Similarly, salmonellosis was reported in the current study in a considerable number of veterinarians (42, 7.9%),

Studies have shown the presence of Mycobacterium bovis in livestock, particularly in cattle, sheep, and goats, in various regions of Saudi Arabia. Al-Hajoj et al. detected M. bovis among cattle and humans, emphasizing the zoonotic transmission potential [33,34]. While the incidence of zoonotic TB in humans is relatively low compared to other forms of TB, it was also reported in the current study that three veterinarians reported previous infection with TB. It has also been documented that cases of human infections with M. bovis in Saudi Arabia were 12 (10-14) per 100,000 in 2015 and it was reduced by 21% during the current year compared to 2015 [34,35].

Leishmania is a parasitic protozoan that causes a group of diseases collectively known as leishmaniasis. In the current study, it was the most reported protozoal infections (29, 5.3%). These diseases can manifest in various forms, including cutaneous, mucocutaneous, and visceral leishmaniasis, depending on the species of Leishmania and the host's immune response. Leishmaniasis is endemic in certain regions of Saudi Arabia, primarily in the southwestern and western parts of the country. It is considered an emerging health problem in Saudi Arabia, with sporadic cases and outbreaks reported [36]. Several Leishmania species have been identified in Saudi Arabia, including Leishmania tropica and Leishmania major. These species are responsible for cutaneous leishmaniasis, the most common form of the disease in the region. Cutaneous leishmaniasis in Saudi Arabia typically presents as skin lesions, which may be single or multiple, and can lead to disfigurement if left untreated. Visceral leishmaniasis is less common but has also been reported in some areas [37,38]. Other infections reported in the current study were only reported in sporadic patterns.

On the other hand, a physical hazard is an agent or extraneous element that exists in the form of an occupational hazard. Contact with animals and equipment, repetitive action, and motor vehicle accidents all pose a risk of physical injury in the practice of veterinary medicine. Most animal bites, scratches, kicks, and crush injuries are caused by improper animal restraint. Lifting, restraining, and treating animals can result in strains, sprains, back injuries, and other repetitive motion injuries [39].

The most serious physical injury to veterinarians is a trauma caused by any bites, scratches, or other injuries sustained when handling the animals for treatment. In addition to acute injuries, veterinarians suffer from repetitive strain injuries or musculoskeletal disorders (MSD), which are inflammatory and degenerative disorders in tendons, muscles, joints, nerves, and blood vessels. Static or awkward postures, repetitive or forceful tasks may be risk factors for the development of MSD in the upper extremities and backbone, and these may be exacerbated by time constraints, work stress, career structure, and after-hours responsibilities [40].

Injuries from sharp instruments such as syringes, needles, scalpel blades, nose tongs for cattle, halters, calf pulling equipment, and metal cattle chutes as well as injuries from falls on slippery surfaces. In this study needle stick injuries constituted about 71.8% in the veterinarians; furthermore, veterinarians may accidentally inject themselves with a needle while uncapping or recapping the needle or while filling the syringe or during vaccination practices. Laboratory workers may be also at risk and these issues should enhance the awareness of using PPE in all occupations related to animals. More likely, the chemical or biological agents introduced at the time of the needle-stick injury caused severe problems; hence, injury from contaminated sharps might result in illness [10].

Animal bites are frequently associated with infections such as rabies. According to rabies monitoring data from the United States, just 7.6% of animal cases are domestic animals [41]. Large animals are most likely to cause injury, predominantly in the upper extremities, but also dog bites, cat bites and scratches, and horse kicks particularly result in dangerous injuries. Other equipment used in veterinary practice such as calf-pulling equipment, metal cattle chutes, restraining equipment, cage doors, ropes, dental drills, hanging scales, and even ophthalmoscopes may cause injury especially to fingers, wrists, and hands [42].

In our study, the most physical injuries were needle sticks followed by animal butting and kicking followed by animal bites (65.4%). These results are in agreement with a study performed by Parmar et al. in India where needle prick was 89.2%, followed by animal kicks (62.8%) and bites (31.8%) [43].

Another type of physical exposure involves ionizing radiation; it is believed that many practicing veterinarians use radiographic equipment. This occurs more frequently in veterinary procedures due to the animals must be restrained and thus the operator may be very close to the source of radiation [44].

Chemical risk can result mainly from the use of gaseous anesthetics, drugs (antineoplastic and antiparasitic agents), detergents, and disinfectants. Many substances used in veterinary practice can cause hazardous effects due to their mutagenic, teratogenic, carcinogenic, and acute toxic nature [2,45]. Chemicals can be swallowed, injected, inhaled, or spilled on the skin by accident; however, veterinarians run the risk of accidentally injecting themselves with things like vaccines, medicines, anesthetics, and animal blood while treating domestic or wild animals. Waste anesthetic gas exposure has been linked to cancer, congenital deformities, spontaneous miscarriages, neurological and psychiatric illnesses, as well as chronic renal and hepatic diseases [2,44].

Detergents, disinfectants, and pesticides are applied directly to animals to control parasites, or they are applied to the area where animals are confined for cleaning and disinfection of premises, equipment, and tools to prevent infectious and contagious diseases, postoperative and iatrogenic infections. They may cause burns by contact and by inhalation leading to inflammation of the mucous membranes of the respiratory tract and ocular-conjunctival irritation and special attention should be paid to items that are poisonous, carcinogenic, or teratogenic. Jeyaretnam et al (2000) suggested that failure to take precautions such as wearing gloves when handling chemicals, could contribute to adverse reactions [4].

Veterinary staff who work outdoors in Saudi Arabia are at risk from many environmental factors such as sun exposure, shed dusts, sandstorms, and heavy rains. Our data showed that many veterinarians are at risk of these environmental factors with the percentages ranging from 83.4% for shed dust to 46.9% for heavy rain. Workers exposed to extreme weather or work in bad environments may be at risk of stress. Extreme weather is a serious condition that can lead to health emergencies in susceptible people, such as those without shelter, outdoor workers, and those working in an area with poor insulation or without heating. These weather-related conditions can lead to serious health problems [46].

Study's limitations

The primary study limitation lies in the use of a cross-sectional design, which restricts our ability to establish temporal or causal associations between the prevalence of occupational hazards and the severity of zoonotic infections, as well as their resulting life-threatening risks. In addition, the respondents did not specify the other biological hazards but merely referred to them as "others."

## Conclusions

In conclusion, the veterinary profession exposes the personnel to a lot of health hazards including biological, physical, chemical, and environmental hazards. There is a need to raise awareness among veterinarians about occupational health risks because they are exposed to many health dangers, especially in the early stages of their careers.
